# Publisher Correction: Two-dimensional Dirac particles in a Pöschl-Teller waveguide

**DOI:** 10.1038/s41598-020-60649-4

**Published:** 2020-03-04

**Authors:** R. R. Hartmann, M. E. Portnoi

**Affiliations:** 1 Physics Department, De La Salle University, 2401 Taft Avenue, Manila, 0922 Philippines; 2 School of Physics, University of Exeter, Stocker Road, Exeter, EX4 4QL United Kingdom; 3 International Institute of Physics, Universidade Federal do Rio Grande do Norte, Natal, RN Brazil

Correction to: *Scientific Reports* 10.1038/s41598-017-11411-w, published online 14 September 2017

This Article contains errors in Reference 10 which was incorrectly given as:

Yldrm, H. & Tomak, M. Nonlinear optical properties of a Pöschl-Teller quantum well. *Phys*. *Rev*. B 72, 115340 (2005).

The correct reference is listed below:

Yildirim, H. & Tomak, M. Nonlinear optical properties of a Pöschl-Teller quantum well. *Phys*. *Rev*. B 72, 115340 (2005).

